# Annexin A1 and the Resolution of Inflammation: Modulation of Neutrophil Recruitment, Apoptosis, and Clearance

**DOI:** 10.1155/2016/8239258

**Published:** 2016-01-13

**Authors:** Michelle Amantéa Sugimoto, Juliana Priscila Vago, Mauro Martins Teixeira, Lirlândia Pires Sousa

**Affiliations:** ^1^Programa de Pós-Graduação em Ciências Farmacêuticas, Faculdade de Farmácia, Universidade Federal de Minas Gerais, 31270-901 Belo Horizonte, MG, Brazil; ^2^Departamento de Análises Clínicas e Toxicológicas, Faculdade de Farmácia, Universidade Federal de Minas Gerais, 31270-901 Belo Horizonte, MG, Brazil; ^3^Laboratório de Imunofarmacologia, Departamento de Bioquímica e Imunologia, Instituto de Ciências Biológicas, Universidade Federal de Minas Gerais, 31270-901 Belo Horizonte, MG, Brazil; ^4^Programa de Pós-Graduação em Biologia Celular, Departamento de Morfologia, Instituto de Ciências Biológicas, Universidade Federal de Minas Gerais, 31270-901 Belo Horizonte, MG, Brazil

## Abstract

Neutrophils (also named polymorphonuclear leukocytes or PMN) are essential components of the immune system, rapidly recruited to sites of inflammation, providing the first line of defense against invading pathogens. Since neutrophils can also cause tissue damage, their fine-tuned regulation at the inflammatory site is required for proper resolution of inflammation. Annexin A1 (AnxA1), also known as lipocortin-1, is an endogenous glucocorticoid-regulated protein, which is able to counterregulate the inflammatory events restoring homeostasis. AnxA1 and its mimetic peptides inhibit neutrophil tissue accumulation by reducing leukocyte infiltration and activating neutrophil apoptosis. AnxA1 also promotes monocyte recruitment and clearance of apoptotic leukocytes by macrophages. More recently, some evidence has suggested the ability of AnxA1 to induce macrophage reprogramming toward a resolving phenotype, resulting in reduced production of proinflammatory cytokines and increased release of immunosuppressive and proresolving molecules. The combination of these mechanisms results in an effective resolution of inflammation, pointing to AnxA1 as a promising tool for the development of new therapeutic strategies to treat inflammatory diseases.

## 1. Introduction

Inflammation is a crucial physiological response for the maintenance of tissue homeostasis, protecting the host against invading microorganisms, foreign substances, or host self-disturbers, such as the molecules derived from damaged cells [1]. After the host has been incited, important microcirculatory events occur in response to local release of proinflammatory mediators, such as histamine, prostaglandins, leukotrienes, cytokines, and chemokines, leading to higher vascular permeability and increased leukocyte recruitment [1]. Leukocytes, such as neutrophils and macrophages, play a key role in inflammatory response, by releasing further inflammatory mediators and acting as effector cells and phagocytes to remove the inflammatory agent/stimuli [2].

Despite the important roles of neutrophils for effective host defense, these cells can also cause tissue damage requiring appropriate regulation [3, 4]. Continuous inflammatory stimuli can lead to aggressive and/or prolonged inflammatory responses, which may be detrimental to the host, leading to chronic inflammation [5]. The efficient removal of the inciting agent by phagocytes is the first signal for triggering proper resolution, through inhibition of proinflammatory mediators production and activation of their catabolism, resulting in the ceasing of further leukocyte recruitment [6]. After that, proresolving pathways are activated in order to restore tissue structure, function, and homeostasis [7, 8]. In this context, anti-inflammatory and proresolving molecules such as specialized lipid mediators (lipoxin A4, resolvins, maresins, and protectins), peptides/proteins (melanocortins, galectins, and annexin A1), and several other substances of different natures are released at the site of inflammation [7, 9, 10]. These endogenous mediators are known for their ability to decrease endothelial activation, reduce leukocyte infiltration, and activate neutrophil apoptosis, which ensures their secure removal by scavenger macrophages through a process called efferocytosis (phagocytosis of apoptotic cells) [4].

**Figure 1:**
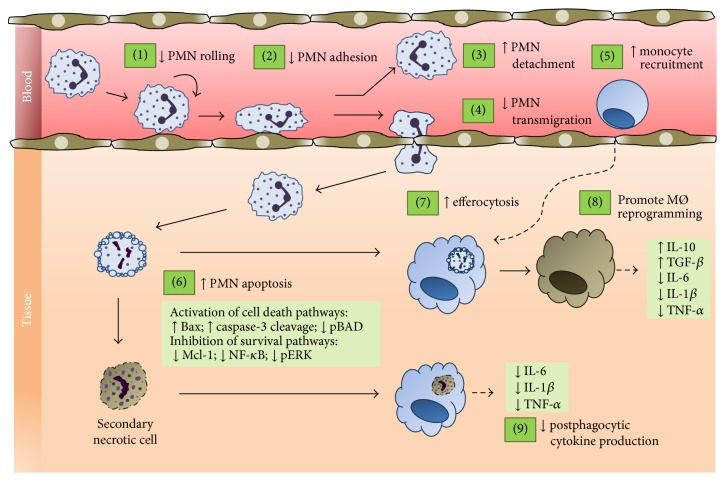
Cellular events associated with the anti-inflammatory and proresolving effects of annexin A1 (AnxA1) and its mimetic N-terminal peptides. AnxA1 modulates a wide range of cellular and molecular steps of the inflammatory response and is deeply involved in the endogenous mechanisms that are activated to bring about proper resolution. Pharmacological administration of AnxA1 results in decreased neutrophil rolling (1) and adhesion (2) to endothelium, increased detachment of adherent cells (3), and inhibition of neutrophil transmigration (4). In addition, AnxA1 is able to induce apoptosis, overriding the prosurvival signals that cause prolonged lifespan of neutrophils at the inflammatory site (6). Endogenous and exogenous AnxA1 also promote monocyte recruitment (5) and clearance of apoptotic neutrophils by macrophages (7). Phagocytosis of apoptotic neutrophils by macrophages is coupled with release of anti-inflammatory signals, including transforming growth factor-*β*, and lower levels of proinflammatory cytokines (8). Besides, AnxA1 is related to macrophage reprogramming toward a proresolving phenotype (8). Initial* in vitro* studies using AnxA1 knock-down leucocytes demonstrate that AnxA1 prevents proinflammatory cytokine production after phagocytosis of secondary necrotic cells. This effect provides an important fail-safe mechanism counteracting inflammatory responses when the timely clearance of apoptotic cells has failed (9).

Annexin A1 (AnxA1) is an important glucocorticoid- (GC-) regulated protein, which contributes to the resolution of inflammation through various ways (Figure 1). AnxA1 limits neutrophil recruitment and production of proinflammatory mediators. Moreover, AnxA1 acts by inducing neutrophil apoptosis, modulating monocyte recruitment, and enhancing the clearance of apoptotic cells by macrophages. Emerging evidence suggests that AnxA1 also induces macrophage reprogramming toward a resolving phenotype, another key event to restore tissue homeostasis. In this review, we summarize several physiological and potential therapeutic actions of AnxA1 on inflammation resolution. In particular, this review highlights recent advances on the actions of this endogenous mediator and its potential clinical utility.

## 2. Annexin A1: General Aspects

Endogenous mediators of inflammation, such as AnxA1, are potential therapeutic tools to control inflammatory diseases. Although whether clinical use of proresolving strategies will be useful for treating inflammatory maladies or will show significant undesirable effects remains to be elucidated, it is believed these will be effective and have fewer side effects due to their ability to mimic or induce natural pathways of the resolution phase of inflammation [8, 12].

Annexin superfamily is composed of 13 members, grouped in view of their unique Ca^2+^-binding-site architecture, which enables them to peripherally attach to negatively charged membrane surfaces [13–15]. AnxA1, also known as annexin I or lipocortin I, was originally identified as a GC-induced protein active on phospholipase- (PL-) A2 inhibition and prevention of eicosanoid synthesis [16–18]. It was subsequently recognized as an endogenous modulator of the inflammatory response, through several studies, mainly those led by Dr. Flower and Dr. Perretti [19, 20]. This 37 kDa protein consists in a homologous core region of 310 amino acid residues, representing almost 90% of the structure, attached to a unique N-terminal region [15]. In addition to mediating membrane binding, Ca^2+^ ions can also induce a conformational change that leads to the exposure of the bioactive N-terminal domain [15, 21, 22]. In fact, studies on the anti-inflammatory activity of AnxA1 revealed not only that the different functions of the protein lie within the unique N-terminus, but also that synthetic peptides from the N-terminal domain may mimic the pharmacological property of the whole protein, specifically binding to formyl peptide receptors (FPRs) [12].

In inflammatory conditions intact AnxA1 (37 kDa) can be cleaved by proteinase-3 and neutrophil elastase generating the 33 kDa cleaved isoform, which is believed to be inactive, and peptides derived from the AnxA1 N-terminus [23–25]. The main cleavage sites on AnxA1 are located at A^11^, V^22^, and V^36^, as identified by cleavage assays coupled to mass spectrometric analyses [25]. Investigation of the AnxA1_2–50_ peptide revealed a novel cleavage site at position 25, probably unmasked due to the simpler conformation of the peptide, compared with the full-length AnxA1 [26]. In fact, the substitution of the mentioned cleavage sites allowed the generation of metabolically stable forms of AnxA1 and its peptide, respectively, named SuperAnxA1 (SAnxA1) [27] and cleavage-resistant AnxA1_2–50_ (CR-AnxA1_2–50_) [26]. The proinflammatory nature of AnxA1 cleavage products is supported by reports of increased levels of the 33 kDa fragment in human and animal inflammatory samples, including bronchoalveolar lavage fluids [28–30] and exudates [11, 25, 31, 32]. For instance, using a model of acute pleurisy, our research group has shown increased levels of the 33 kDa breakdown product of AnxA1 during the time points of high neutrophil infiltration into the pleural cavity followed by regain of the intact form during the resolving phase of the pleurisy [11]. However, what the biological functions of this and other AnxA1-generated peptides are is still unclear, and this matter deserves further investigation.

Evidence for physiological function of AnxA1 in modulating inflammation emerged from studies involving AnxA1-null mice and AnxA1 neutralization strategies. AnxA1-null mice are viable and have a normal phenotype until they are challenged with inflammatory stimuli when they show stronger and more prolonged inflammatory reaction when compared to the wild-type (WT) [33–40]. Resistance to glucocorticoid treatment and aberrant inflammation in AnxA1-deficient mice provided initial evidence for the physiological relevance of the protein [33]. In the absence of AnxA1, the inflammatory response is exacerbated as demonstrated by increased neutrophil extravasation following zymosan-induced peritonitis [35] and endotoxin-induced uveitis [37]. In addition, animals lacking this protein exhibited exacerbated arthritis severity [34] and allergic response in ovalbumin-induced conjunctivitis [39]. AnxA1 KO mice also showed increased atherosclerotic lesion size with an overall increase in lesional macrophages and neutrophils [40]. Moreover, our research group has shown the prevention of spontaneous and dexamethasone-driven resolution of inflammation by using an AnxA1 neutralizing strategy [11]. Aside from the physiological role of the endogenous protein, pharmacological treatment with both human recombinant AnxA1 and its N-terminal peptides exerts anti-inflammatory and proresolving effects in a variety of experimental models, highlighting their therapeutic potential for inflammation resolution [11, 26, 27, 41] and wound repair [42].

AnxA1 exerts many of its anti-inflammatory and proresolving actions through the formyl peptide receptor type 2/lipoxin A4 receptor (FPR2/ALX). This receptor, along with FPR1 and FPR3, composes a family of seven-transmembrane domain G protein-coupled receptors which share significant sequence homology [43]. FPR2/ALX receptor is shared by a variety of other peptide/protein and lipid ligands, mediating diverse biological functions of relevance for host defence and inflammation. Interestingly, FPR2/ALX agonists are associated with both proinflammatory (e.g., serum amyloid A and cathelicidin) and proresolving (e.g., AnxA1 and LXA_4_) signalling pathways [43, 44]. However, how FPR2/ALX can promote both inflammatory response and limit its duration and intensity still remains to be fully elucidated. It is noteworthy that distinct FPR2/ALX domains are required for signalling by different agonists [45]. Using FPR2/ALX transfected cells and chimeric FPR1 and FPR2 clones, Bena and col. (2012) identified that while AnxA1-mediated signalling involves the N-terminal region and extracellular loop II of FPR2/ALX, SAA interacts with the extracellular loops I and II of the same receptor [45]. Otherwise, LXA_4_ has been shown to activate FPR2/ALX by interacting with extracellular loop III and the associated transmembrane domain [46].

The versatility of FPR2/ALX receptors also seems to rely on the activation of receptor dimmers in a biased fashion. AnxA1 was found to activate FPR2/ALX homodimerization but not the proinflammatory SAA [47]. In contrast to the full-length AnxA1, the short AnxA1 derived peptide Ac2–26 is able to activate all members of the human FPR family [48] and induce FPR2/ALX-FPR1 heterodimerization [47]. These observations suggest that short AnxA1 mimetic peptides might fulfill other functions at variance to those reported for the parental protein [49]. However, a good degree of selectivity was retained by longer AnxA1 derived anti-inflammatory sequences such as AnxA1_2–50_ [26].

Interestingly, the promiscuity of FPR2/ALX seems to be linked to a network of resolution mediators as discussed by Brancaleone and col. (2011) [50]. In fact, the authors provide strong evidence that the engagement of FPR2/ALX by selective agonists (such as LXA_4_ and antiflammin 2) would induce AnxA1 phosphorylation and mobilization in human PMN [50]. In a similar vein, the proresolving mediator Resolvin E1 (RvE1) stimulates endogenous LXA_4_ production [51, 52]. Moreover, it has been shown that proresolving mediators such as resolvins and LXA_4_ induce further anti-inflammatory molecules* in vivo*, such as interleukin- (IL-) 10 [41]. Taken together, these data suggest that a proresolving cascade may be operating during resolution with FPR2/ALX playing a central role in this process.

## 3. Anti-Inflammatory and Proresolving Actions of AnxA1

### 3.1. AnxA1 Regulates Neutrophil Recruitment to the Inflammatory Site

During inflammation neutrophils are rapidly recruited to the infected or injured tissue. However, due to the potential tissue-damaging effects of PMN, their fine-tuned regulation at the inflammatory site is required [53]. Indeed, exacerbated or overshooting inflammatory response with high neutrophil influx may account for chronic inflammatory diseases [5]. Thus, restricting leukocyte infiltration to the tissue is an essential process for spontaneous or pharmacological-induced resolution of inflammation [4, 8].

Neutrophil trafficking to the site of inflammation requires adhesion and transmigration through blood vessels, which is orchestrated by molecules on leukocytes (e.g., *β*1, *β*2 integrins, and L-selectin) and on endothelial cells (e.g., vascular cell adhesion molecule-1, intercellular adhesion molecule-1, and E-selectin). The leukocyte adhesion cascade is a tightly regulated process, subjected to both positive and negative regulators [71]. For example, anti-inflammatory and proresolving mediators, such as AnxA1, are well documented to counterregulate excessive neutrophil accumulation (an anti-inflammatory action). Human PMN interaction with endothelial cells during the early stage of inflammation promotes modulation of AnxA1 in several ways, such as induction of gene expression [35] and mobilization and cell surface externalization of intracellular AnxA1 [72, 73]. In turn, the externalized protein acts as a brake for PMN adhesion to the microvascular wall, preventing overexuberant cell transmigration to the inflammatory site [4, 27, 72, 74]. Dalli and col. (2008) [75] reinforced the anti-inflammatory properties of PMN-derived microparticles containing functionally active AnxA1. Released upon adhesion to endothelial cells, these microparticles inhibit neutrophil/endothelium interaction under flow,* in vitro*, and PMN recruitment to an air pouch inflamed with IL-1*β*,* in vivo *[75]. Moreover, microparticles derived from WT but not from AnxA1-deficient neutrophils were able to inhibit IL-1*β*-induced leukocyte trafficking [75].

Several studies using exogenously administrated AnxA1 have provided further evidence for the modulating role of AnxA1 on neutrophil trafficking.* In vivo* observations produced through intravital microscopy techniques indicated that AnxA1 and Ac2–26 administration to mice during zymosan-induced peritonitis produced detachment of adherent neutrophils from the vascular wall with consequent inhibition of neutrophil extravasation across mouse mesenteric postcapillary venules (Table 1) [56]. Supporting these first findings,* in vitro* studies showed that recombinant AnxA1 and its mimetic peptides display inhibitory effects on neutrophil rolling [26, 27, 54, 55] adhesion to endothelial monolayer [26, 27, 40, 48, 54, 55] and transmigration [48].

Shedding of L-selectin appears to be one of the molecular mechanisms that mediate the effects of AnxA1 and its N-terminal peptides on neutrophil recruitment. Walther and col. (2000) [48] have described the ability of the AnxA1 peptide Ac9–25 to cause transient calcium fluxes and L-selectin shedding in human neutrophils. After that, the same mechanism was linked to the inhibitory effects of Ac2–26 on PMN capture and rolling in a flow chamber assay [54]. Similarly, promotion of L-selectin shedding was demonstrated for human recombinant AnxA1 [57, 58], an effect mediated by cell surface metalloprotease (“sheddase”) [58]. Recently, Drechsler and col. (2015) [40] brought further insights into the mechanisms behind the antimigratory effects of Ac2–26. According to the authors, the peptide dose dependently reduces the affinity of activated neutrophils for vascular cell adhesion molecule-1 (VCAM-1) and intercellular adhesion molecule-1 (ICAM-1), a response abrogated in cells harvested from FPR2 knockout mice. They demonstrated that Ac2–26 inhibits the adhesiveness of *β*1 and *β*2 integrins by downmodulating their affinity and valency, but without changing their cell surface expression. It was also demonstrated that Ac2–26 interferes with the chemokine-driven activation of Rap1, an essential step in integrin activation [76, 77].

Pederzoli-Ribeil and col. (2010) [27] combined* in vitro* and* in vivo* experimental strategies to show that AnxA1 and its mutant cleavage-resistant form, SAnxA1, are able to augment rolling velocity and reduce adhesion of PMN to endothelial cells through FPR2 receptors. Furthermore, Dalli and col. (2013) [26] demonstrated the anti-inflammatory actions of the longer acetylated AnxA1 peptide AnxA1_2–50_ and its cleavage-resistant form, CR-AnxA1_2–50_. Both displayed antimigratory effects* in vivo*, reducing leukocyte adhesion to inflamed cremaster venule, neutrophil migration into dermal air pouches in response to IL-1*β*, and neutrophil migration into peritoneum in response to zymosan.

*In vivo* anti-inflammatory and antimigratory properties of the short AnxA1 peptide Ac2–26 have also been extensively demonstrated, as exemplified by its ability to inhibit carrageenan-induced PMN adhesion to the vasculature and extravasation into the peritoneal fluid [74]. The peptide was also able to prevent neutrophil recruitment in myotoxin-induced peritonitis [78] and during lung inflammation induced by intestinal ischemia/reperfusion [79]. Moreover, Ac2–26 showed potential benefits in an ocular model by inhibiting neutrophil influx, protein leak, chemical mediator release, and COX-2 expression during endotoxin-induced uveitis [37]. The Ac2–26 peptide also demonstrated antimigratory effects in a model of ovalbumin-induced allergic conjunctivitis, significantly reducing the clinical signs of conjunctivitis through the inhibition of leukocyte influx and cytokines and chemokines release, effects correlated with inhibition of the ERK pathway [39]. Interestingly, increased levels of ERK phosphorylation were associated with exacerbated allergic response observed in AnxA1-deficient mice in comparison to WT animals [39]. Reinforcing the involvement of AnxA1 pathway in neutrophil recruitment, AnxA1-null mice demonstrated a higher extent of neutrophil extravasation in animal models of peritonitis [35, 74], allergic conjunctivitis [39], and uveitis [37].

AnxA1 may also be tightly coupled to the anti-inflammatory properties of other FPR2/ALX agonists such as LXA_4_ and antiflammin 2 (AF-2) [50]. The nonapeptide AF-2, which corresponds to region 246–254 of AnxA1 [80], is known to interfere with PMN activation, chemotaxis, and adhesion to endothelial cells [60, 61], via FPR2/ALX receptor [81]. Also, LXA_4_ is a potent regulator of PMN trafficking in experimental inflammation [9, 82]. Interestingly, recent data indicated a crucial role for endogenous AnxA1 in the detachment phenomenon promoted by both compounds [50]. For instance, LXA_4_ and AF-2 lost their antimigratory effects in AnxA1 KO mice suggesting AnxA1 as a downstream mediator of other proresolving and anti-inflammatory molecules [50].

### 3.2. AnxA1 Induces Neutrophil Apoptosis

Neutrophils are produced in the bone marrow from myeloid stem cells, which in turn proliferate, differentiate into mature neutrophils, and are delivered into circulation [83]. Although the circulatory half-life of neutrophils is now thought to be longer than previously estimated (days instead of hours) [84], at inflammatory sites the constitutive apoptotic pathway is delayed by the action of local inflammatory mediators, resulting in increased neutrophil half-life [85], an effect that can be opposed by proresolving mediators including AnxA1 and lipoxins [86].

**Figure 2:**
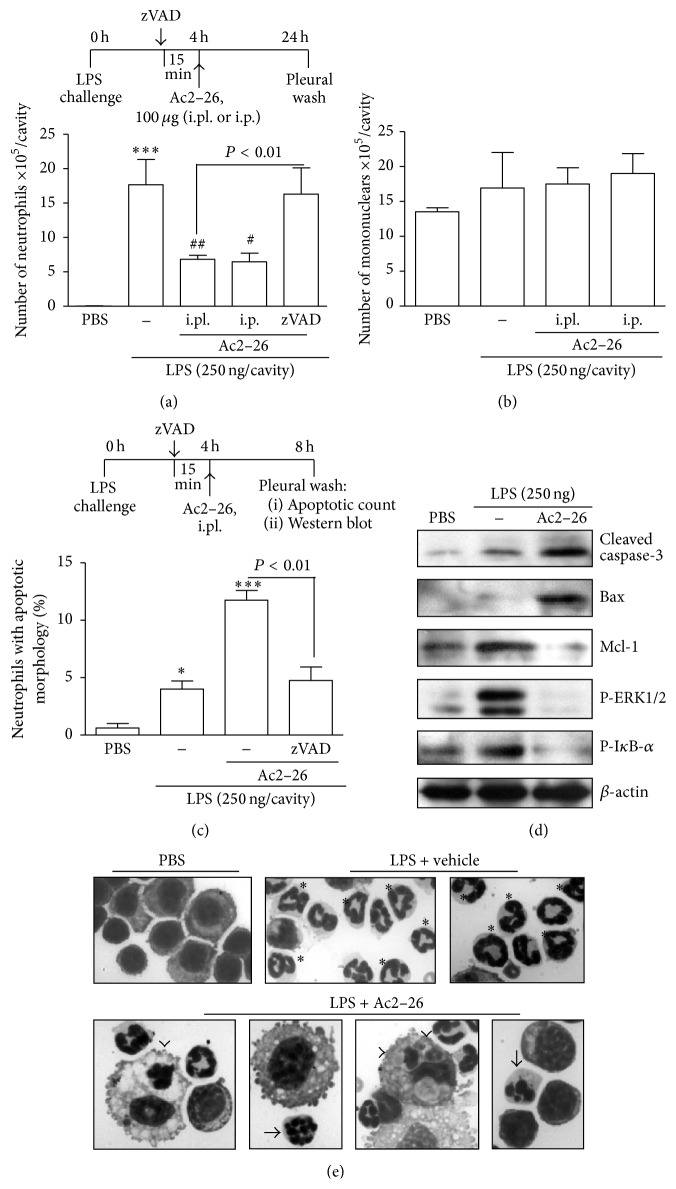
Effect of exogenous administration of AnxA1 derived peptide Ac2–26 on LPS-induced pleurisy. Mice were injected with LPS (250 ng/cavity, i.pl.) and 4 h later received an injection of Ac2–26 (100 *μ*g/mouse, i.pl. or i.p.). The treatment with the pan-caspase inhibitor zVAD-fmk (1 mg/kg, i.p.) was performed 15 min before the injection of peptide. The numbers of neutrophils (a) and mononuclear cells (b) were evaluated 20 h after drug treatment. Cells with distinctive apoptotic morphology (c and e) and Western blot for detection of cleaved caspase-3, Bax, Mcl-1, P-ERK, and P-I*κ*B-*α* (d) were evaluated 4 h after the peptide treatment. ^*∗*^ *P* < 0.05 or ^*∗∗∗*^ *P* < 0.001 when compared with PBS-injected mice and ^#^ *P* < 0.05 or ^##^ *P* < 0.01 when compared with vehicle-treated, LPS-injected mice. (e) Representative figures of nonapoptotic (asterisk) and apoptotic (arrows) neutrophils and apoptotic cells inside macrophages (arrowheads). PBS and vehicle (upper panels) and Ac2–26-treated (lower panels) animals are shown. Original data from Vago et al., 2012 [11].

In addition to affecting the migration of leukocytes through FPR activation, strong evidence of the involvement of AnxA1 on neutrophil apoptosis has emerged. Proapoptotic effect of AnxA1 on neutrophils was first described* in vitro* associated with transient calcium fluxes and dephosphorylation of BAD, an intracellular protein whose proapoptotic function is lost upon phosphorylation [57]. Our group [11] demonstrated the* in vivo *proapoptotic functions of endogenous AnxA1 during self-resolving inflammation. In an acute pleurisy model, blockage of the AnxA1 pathway by using a specific anti-AnxA1 antiserum prevented dexamethasone- (dexa-) induced resolution of neutrophilic inflammation, abolishing morphological and biochemical apoptotic events in the pleural cavity. AnxA1 neutralization also hampered dexa-induced decrease of ERK1/2 and I*κ*B-*α* phosphorylation and Bax accumulation. In addition, anti-AnxA1 treatment prevented spontaneous resolution of neutrophilic inflammation, suggesting an important role of endogenously produced AnxA1 in the proresolutive program [11]. Furthermore, pharmacological administration of Ac2–26 peptide promoted active resolution and augmented the extent of neutrophil apoptosis. These effects were prevented by the pan-caspase inhibitor zVAD-fmk and linked to activation of the cell death pathways Bax and caspase-3 and inhibition of the survival-controlling pathways Mcl-1, ERK1/2, and NF-*κ*B [11] (Figure 2).

In a skin allograft model, pharmacological treatment with Ac2–26 increased transplantation survival related to inhibition of neutrophil transmigration and induction of apoptosis, thereby reducing the tissue damage compared with control animals [62].* In vitro*, Ac2–26 counteracted the survival signal in SAA-treated neutrophils, an effect associated with caspase-3 cleavage and prevented by the JNK inhibitor [47]. Dalli and col. (2013) also demonstrated that AnxA1_2–50_ and CR-AnxA1_2–50_ peptides can override the antiapoptotic effect of SAA in human neutrophils* in vitro *[26]. This proapoptotic effect may have contributed to the* in vivo* anti-inflammatory and proresolving actions of the peptides characterized by reduced granulocyte counts and enhanced efferocytosis in peptide-treated mice during peritonitis [26].

AnxA1 has also been described as a mediator of drug-induced apoptosis, supporting its involvement in the induction of cell death. The proapoptotic effect described for the histone deacetylase inhibitor (HDCAI) FK228, in leukemia cells, was linked to the induction of AnxA1 expression, externalization, and cleavage. Neutralization with anti-AnxA1 antibody or gene silencing with AnxA1 siRNA inhibited FK228-induced apoptosis, suggesting the involvement of AnxA1 in apoptotic cell death in response to HDCAI [87]. Recently, the* in vitro* ability of HDACIs to promote apoptosis was also demonstrated in bone-marrow neutrophils from WT but not from AnxA1 knockout mice [88].* In vivo*, HDACIs significantly reduced neutrophil numbers and induced neutrophil apoptosis in a zymosan-induced peritonitis model. Once again, the lack of AnxA1 hampered this* in vivo* proapoptotic effect [88].

It is important to keep in mind that the proapoptotic effect of AnxA1 can be underestimated in dynamic* in vivo* models of inflammation. Regarding other anti-inflammatory drugs, it is documented in a number of diverse experimental and clinical settings that small changes in apoptosis rates can promote dramatic changes in total neutrophil numbers over time. This observation is most likely due to rapid recognition and phagocytosis of apoptotic cells [89–91].

### 3.3. AnxA1 Induces Monocyte Recruitment and Increases Efferocytosis

Macrophage phagocytic clearance of apoptotic neutrophils plays an important role in the resolution of inflammation since this process prevents excessive neutrophil activation and the exposure of tissues to noxious neutrophil intracellular contents [92, 93]. For this reason, appropriate (nonphlogistic) monocyte recruitment from the bloodstream to inflammatory sites is a critical step in acute inflammation, enabling the clearance of apoptotic neutrophils and orderly progression towards resolution.

It has long been established that extravasation of PMN to the site of inflammation contributes to the launch of monocyte recruitment, with PMN granule proteins being important monocyte attractors [94]. Recent research from Perretti's group [64] indicates apoptotic neutrophils as the principal reservoir of AnxA1, which acts as important recruiting agent for monocytes to orchestrate the second resolving phase of acute inflammation. Associating* in vitro* and* in vivo* experiments, Professor Mauro Perretti's group filled an important gap in our knowledge by demonstrating the central role of the AnxA1–ALX/FPR2 pathway in modulating monocyte recruitment [64]. The authors demonstrated that intraperitoneal administration of AnxA1 induced monocyte migration, an effect absent in FPR2 null mice. Supporting these findings, both AnxA1 and FPR2/ALX null mice challenged with intraperitoneal zymosan exhibited diminished recruitment of monocytes as compared to WT mice, despite the higher levels of chemoattractants [64].

After initial steps of apoptosis, neutrophils lose their functional properties, such as the ability to move by chemotaxis, generate a respiratory burst, or degranulate [95]. Furthermore, they exhibit alterations on their intracellular pathways and cell surface molecules while some externalized molecules, such as phosphatidylserines (PS), facilitate the recognition and removal of apoptotic neutrophils by macrophages [92, 96].

Recent studies have reported that AnxA1 from apoptotic cells is involved in their phagocytic clearance. The first observation that AnxA1 participates in the engulfment of apoptotic cells was described by Arur and col. (2003) [97]. By using a differential proteomics technology, they showed that AnxA1 is exported to the outer plasma membrane of apoptotic lymphocytes, colocalizes with PS, and is required for efficient clearance of apoptotic cells, suggesting a role for AnxA1 as bridging PS molecules on apoptotic cells to phagocytes [97]. Scannell and col. (2007) [65] subsequently demonstrated that apoptotic neutrophils release AnxA1, which acts on macrophages, promoting the removal of effete cells [65]. Noteworthily, not only the intact form of AnxA1 released by apoptotic cells but also the cleavage fragments, under 10 kDa, were effective in stimulating efferocytosis [65].

Studies have also documented macrophages as a source of endogenous AnxA1, which in turn facilitates phagocytic uptake of apoptotic cells. Maderna and col. (2005) showed that human macrophages release AnxA1 upon treatment with GC and that this protein acts in autocrine or paracrine manners to increase the engulfment of apoptotic neutrophils [67]. Additional experiments with AnxA1-null mice provided further evidence for a functional role of AnxA1 in efferocytosis, as macrophages derived from their bone marrow were defective in clearance of apoptotic cells [67]. In fact, the authors demonstrated,* in vitro*, the ability of the AnxA1 mimetic peptide Ac2–26 to promote phagocytosis of apoptotic PMN by human macrophages, an effect associated with actin rearrangement in the phagocytic cells and abrogated in the presence of FPR antagonist [67]. Subsequently, it was clearly demonstrated the nonredundant function of FPR2/ALX receptor in Ac2–26 induced efferocytosis since the peptide failed to exert its proefferocytic action on FPR2/ALX deficient macrophages [98]. Furthermore, Yona and coworkers (2006) associated* in vitro *and* in vivo* strategies that indicated reduced phagocytosis of zymosan particles by AnxA1 knockout macrophages [99].

It has been proposed that AnxA1 released by macrophages can opsonize apoptotic cells, probably by interacting with surface-exposed PS, enhancing their uptake by phagocytes [66]. Interestingly, McArthur's group demonstrated that the binding of microglial-derived AnxA1 to PS on the surface of apoptotic neuronal cells is critically required for phagocytosis [69]. Moreover, Dalli and colleagues (2012) reported that AnxA1 expressed by resident macrophages is a critical determinant for the clearance of senescent neutrophils in the bone marrow [100]. Proefferocytic effects were also observed for AnxA1_2–50_ and its cleavage-resistant form (CR-  AnxA1_2–50_), which stimulated efferocytosis* in vitro* by human and mice bone-marrow derived macrophages [26]. This effect was confirmed* in vivo *in a zymosan-induced peritonitis model, when the peptides significantly reduced exudate neutrophil counts and increased the number of macrophages containing ingested PMN [26].

Once phagocytic removal of apoptotic cells has failed, neutrophils undergo secondary postapoptotic necrosis, probably leading to the leakage of cytotoxic and antigenic intracellular contents into the surrounding tissue [63]. Blume and col. (2012) revealed, in two complementary studies, the role of externalized AnxA1 as a fail-safe mechanism after neutrophil transition from apoptosis to secondary necrosis. First, they described AnxA1 externalization during secondary necrosis, which in turn promotes the removal of dying cells and prevents proinflammatory cytokine production [66]. In the second study, they demonstrated that* in vitro* AnxA1 proteolysis during secondary necrosis generates a monocytic “find-me” signal, contributing to the recruitment of monocytes and consequently preventing inflammation [63].

The removal of apoptotic cells has dual importance: prevention of potentially toxic content release and induction of macrophage reprogramming toward a resolving phenotype [101–103], another key event to restore tissue homeostasis. Accordingly, AnxA1-induced efferocytosis is coupled with increased release of transforming growth factor- (TGF-) *β* and lower levels of the proinflammatory cytokine IL-6 [65, 67]. In agreement with this observation, impaired phagocytosis in AnxA1-deficient macrophages is mirrored by increased release of tumor necrosis factor- (TNF-) *α* and IL-6 [99]. Supporting an immunomodulatory effect of AnxA1 on cytokine production, AnxA1-null mice showed increased mortality in a model of LPS-induced endotoxic shock which was correlated with increased activation of inflammatory cells [104]. The authors detected delayed and more prolonged increase in the levels of TNF-*α*, IL-1, and IL-6 in the blood of AnxA1-null mice, as well as increased production of these cytokines by AnxA1 KO macrophages [104]. This data is consistent with the increased production of IL-6 and TNF by stimulated AnxA1 KO peritoneal macrophages in comparison to WT cells [105]. Moreover,* in vitro* studies linked AnxA1 to brain homeostasis, demonstrating that exogenous AnxA1 can suppress microglial activation, limiting indiscriminate phagocytosis of healthy neurones and nitric oxide (NO) production during the phagocytic reaction [69]. Recently, the functional role of macrophage-derived AnxA1 in modulating hepatic inflammation and fibrogenesis during nonalcoholic steatohepatitis (NASH) progression was documented [68]. NASH in AnxA1 KO mice was characterized by enhanced lobular inflammation resulting from increased macrophage recruitment and exacerbation of the proinflammatory M1 phenotype [68]. In line with these results, AnxA1 administration to liver macrophages suppressed M1 activation, characterized by reduced expression of iNOS and IL-12p40, and increased IL-10 expression. Interestingly, activation of FPR2 by AnxA1 skewed M1 macrophages to anti-inflammatory M2-like cells, attenuating the expression of IL-6, IL-1*β*, and TNF-*α* [44]. Furthermore, Cooray and col. (2013) revealed an AnxA1-specific FPR2/ALX proresolving signal pathway centered in p38, leading to the production of IL-10 by human monocytes, an effect replicated* in vivo* after intraperitoneal AnxA1 injection [47].

Although uptake of secondary necrotic leukocytes was shown to be AnxA1 independent, the protein has an anti-inflammatory action on macrophages, since phagocytosis of AnxA1 knock-down necrotic cells induced increased release of proinflammatory cytokines TNF, IL-6, and IL-1*β* by phagocytic cells [66]. Pupjalis and col. (2011) added knowledge to the immunosuppressive actions of AnxA1 derived from apoptotic PMN. According to the authors, the treatment of human monocytes with AnxA1-containing supernatant of apoptotic granulocytes or Ac2–26 peptide results in a significantly diminished release of proinflammatory cytokines when the monocytes are subsequently challenged with endotoxin [70].

Taken together, these findings indicate that AnxA1-induced efferocytosis collaborates with the resolution of inflammation by promoting the elimination of effete neutrophils allied to an alternative macrophage activation that downregulates the production of proinflammatory mediators. Such events pave the way to the resolution of inflammation.

## 4. Concluding Remarks

AnxA1 is a GC-regulated protein that modulates a wide range of cellular and molecular steps of the inflammatory response and is deeply involved in the endogenous mechanisms that are activated to bring about proper resolution. So, it is reasonable to suppose that AnxA1-based pharmacologic strategies could be as effective as steroids, without their metabolic side effects. We have discussed here the ability of AnxA1 and its mimetic peptides to limit neutrophil accumulation in the tissue. Besides limiting neutrophil recruitment and increasing neutrophil apoptosis, AnxA1 promotes apoptotic neutrophil clearance by modulating monocyte recruitment and enhancing efferocytosis. Indeed, AnxA1 contributes to tissue homeostasis by inducing macrophage reprogramming toward a resolving phenotype. The combination of these mechanisms results in an effective resolution of inflammation, pointing to AnxA1 and its mimetic peptides as promising therapeutic agents for treating inflammatory diseases.

The promising findings on the potential therapeutic use of AnxA1 in inflammatory diseases have stimulated the development of pharmaceutical formulations containing AnxA1 mimetic peptides, such as the controlled-release hydrogels for dermal wound repair application [106] and targeted polymeric nanoparticles [107]. The latter demonstrated ability to enhance resolution in zymosan-induced peritonitis [107], promote colonic wounds healing [42], and protect hypercholesterolemic mice against advanced atherosclerosis [108]. These pharmaceutical strategies offer further benefits, overcoming the critical pharmacokinetics of short peptides* in vivo*, protecting them from proteolysis during pharmacological treatment, and facilitating the delivery to injury sites.

## Figures and Tables

**Table 1:** *In vitro* and *in vivo* evidence for anti-inflammatory and proresolving properties of annexin A1 and its fragments.

Agent	Experimental model	Outcome/effect on resolution	References
*Inhibition of neutrophil recruitment*			

AnxA1	Neutrophil/endothelial interaction (*in vitro*)	↓ PMN capture, rolling, and adhesion ↓ PMN transmigration	[27, 48, 54, 55]
	Neutrophil/endothelial interaction (*in vivo*)	↓ PMN rolling, adhesion, and emigration ↑ Detachment of adherent PMN	[27, 56]
	Human PMN	↑ L-selectin shedding	[57, 58]
	IL-1*β* inflamed air pouch	↓ PMN migration	[26, 59]
	Carrageenan-induced paw edema	↓ edema ↓ leukocyte infiltration	[27]

SAnxA1	Neutrophil/endothelial interaction (*in vitro*)	↓ PMN capture, rolling, and adhesion	[27]
	Neutrophil/endothelial interaction (*in vivo*)	↓ PMN rolling and adhesion	[27]
	fMLP induced skin edema	↓ MPO activity	[27]
	Carrageenan-induced paw edema	↓ edema ↓ leukocyte infiltration	[27]

AnxA1_2–50_	Neutrophil/endothelial interaction (*in vitro*)	↓ PMN rolling and adhesion	[26]
	Neutrophil/endothelial interaction (*in vivo*)	↓ PMN adhesion	[26]
	IL-1*β* inflamed air pouch	↓ PMN recruitment	[26]

Ac2–26	Neutrophil/endothelial interaction (*in vitro*)	↓ PMN capture, rolling, and adhesion ↑ L-selectin shedding	[40, 54]
	Human PMN activated with CCL5	↓ *β* integrin activation	[40]
	Neutrophil/endothelial interaction (*in vivo*)	↓ PMN adhesion and emigration ↑ detachment of adherent PMN	[56]

Ac1–26	Neutrophil/endothelial interaction (*in vitro*)	↓ PMN transmigration	[48]

Ac9–25	Neutrophil/endothelial interaction (*in vitro*)	↓ PMN adhesion and transmigration ↑ L-selectin shedding	[48]

AF-2	Neutrophil/endothelial interaction (*in vitro*)	↓ PMN adhesion ↓ *β*2 integrin expression	[60, 61]

*Induction of neutrophil apoptosis*			

AnxA1	Human PMN	↑ apoptosis (↓ pBAD)	[57]

AnxA1_2–50_	Human neutrophils stimulated with SAA	↑ apoptosis	[26]

Ac2–26	Human neutrophils stimulated with SAA	↑ apoptosis (↑ caspase-3 cleavage; JNK dependent)	[47]
	Acute pleurisy	↑ apoptosis (↑ Bax; ↑ caspase-3 cleavage; ↓ Mcl-1; ↓ NF-*κ*B; ↓ pERK)	[11]
	Skin allograft model	↑ skin allograft survival ↑ apoptosis ↓ neutrophil transmigration	[62]

*Enhancement of monocyte recruitment and efferocytosis *			

Ac2–7	Transmigration assay (*in vitro*)	Stimulating human monocyte chemotaxis	[63]

AnxA1	Chemotaxis assays	Human monocyte chemoattractant	[64]
	Administration to mouse peritoneum	↑ monocyte recruitment	[64]
	Phagocytosis of apoptotic leukocytes	↑ efferocytosis ↑ binding of apoptotic cells to MØ	[65, 66]

Ac2–26	Phagocytosis of apoptotic neutrophils	↑ phagocytosis Inducing actin reorganization ↑ TGF-*β* release ↓ IL-8 release	[67]

AnxA1_2–50_	Zymosan-induced peritonitis	↑ efferocytosis	[26]

*Macrophage reprogramming*			

AnxA1	Human MØ cell line	Induced M2-like polarization	[44]
	Human monocytes	↑ IL-10	[47]
	LPS stimulated THP-1 MØ	↓ IL-6, TNF, and IL-1*β*	[66]
	MØ from NASH livers	↓ M1 polarization (↓ iNOS, IL-12p40) ↑ IL-10	[68]
	Intraperitoneal injection	↑ IL-10	[47]
	Phagocytosis of apoptotic neurons by microglial cells	↓ phagocytosis of healthy cells ↓ NO production	[69]

Ac2–26	Endotoxin-challenged monocytes	↓ IL-6 signalling ↓ TNF-*α* release	[70]

AnxA1: annexin A1; fMLP: N-Formyl-Met-Leu-Phe; IL: interleukin; MPO: Myeloperoxidase; MØ, macrophage; NASH; nonalcoholic steatohepatitis; PMN: polymorphonuclear; NO: nitric oxide; SAA: serum amyloid A; SAnxA1: SuperAnxA1 (proteinase-3 resistant); TGF-*β*: transforming growth factor-*β*; TNF-*α*: tumor necrosis factor alpha.

## Abbreviations

Ac2–26: Peptide from N-terminal portion of annexin A1 (residues 2–26)
AnxA1: Annexin A1
FPR2/ALXR: Formyl peptide receptor 2/lipoxin A4 receptor
Boc-1: N-t-Boc-Met-Leu-Phe
Dexa: Dexamethasone
ERK1/2: Extracellular signal-regulated kinase
FPR: Formyl peptide receptor
GC: Glucocorticoid
ICAM-1: Intercellular adhesion molecule-1
i.pl.: Intrapleural
IL: Interleukin
I*κ*B-*α*: Inhibitory kappa B alpha
KO: Knockout
LPS: Lipopolysaccharide
Mcl-1: Myeloid cell leukemia-1
NF-*κ*B: Nuclear factor kappa B
PMN: Polymorphonuclear
SAnxA1: SuperAnxA1 or cleavage-resistant AnxA1
TGF-*β*: Transforming growth factor-*β*
TNF: Tumor necrosis factor
VCAM-1: Vascular cell adhesion molecule-1
WT: Wild-type
zVAD-fmk: Benzyloxycarbonyl-Val-Ala-Asp-fluoromethylketone.
